# Performance Evaluation of 3 Large Language Models for Nutritional Content Estimation from Food Images

**DOI:** 10.1016/j.cdnut.2025.107556

**Published:** 2025-09-09

**Authors:** Jonatan Fridolfsson, Emma Sjöberg, Meri Thiwång, Stefan Pettersson

**Affiliations:** 1 Center for Lifestyle Intervention, Institute of Medicine, University of Gothenburg, Gothenburg, Sweden; 2 Department of Medicine Geriatrics and Emergency Medicine Östra, Sahlgrenska University Hospital, Gothenburg, Sweden; 3 Center for Health and Performance, Department of Food and Nutrition and Sport Science, University of Gothenburg, Gothenburg, Sweden

**Keywords:** dietary assessment, large language model, artificial intelligence, validation study, ChatGPT, Claude, Gemini

## Abstract

**Background:**

Traditional dietary assessment methods face limitations including recall bias, participant burden, and portion size estimation errors. Recent advances in artificial intelligence, particularly multimodal large language models (LLMs), offer potential solutions for automated nutritional analysis from food images.

**Objectives:**

This study aims to evaluate and compare the performance of 3 leading LLMs (ChatGPT-4o, Claude 3.5 Sonnet, and Gemini 1.5 Pro) in estimating food weight, energy content, and macronutrient composition from standardized food photographs.

**Methods:**

We analyzed 52 standardized food photographs including individual food components (*n* = 16) and complete meals (*n* = 36) in 3 portion sizes (small, medium, large). Each model received identical prompts to identify food components and estimate nutritional content using visible cutlery and plates as size references. Model estimates were compared against reference values obtained through direct weighing and nutritional database analysis (Dietist NET). Performance metrics included mean absolute percentage error (MAPE), Pearson correlations, and systematic bias analysis using Bland–Altman plots.

**Results:**

ChatGPT and Claude demonstrated similar accuracy with MAPE values of 36.3% and 37.3% for weight estimation, and 35.8% for energy estimation. Gemini showed substantially higher errors across all nutrients (MAPE 64.2%–109.9%). Correlations between model estimates and reference values ranged from 0.65 to 0.81 for ChatGPT and Claude, compared with 0.58–0.73 for Gemini. All models exhibited systematic underestimation that increased with portion size, with bias slopes ranging from –0.23 to –0.50.

**Conclusions:**

ChatGPT and Claude achieved accuracy levels comparable with traditional self-reported dietary assessment methods but without associated user burden, suggesting potential utility as dietary monitoring tools. However, systematic underestimation of large portions and high variability in macronutrient estimation indicate these general-purpose LLMs are not yet suitable for precise dietary assessment in clinical or athletic populations where accurate quantification is critical.

## Introduction

Accurate dietary assessment is fundamental for understanding nutritional intake and health outcomes. Current methods include retrospective 24-hour recalls and prospective dietary records; weighed food records (WFR) and estimated diet records (EDR). Although WFR is considered the reference standard, it increases participant burden and can alter eating behavior [1]. EDR is more practical but introduces substantial measurement errors, particularly for portion size estimation in mixed or cooked meals where weight changes occur through water absorption or loss [2]. Studies show systematic biases with individuals underreporting energy-dense foods while overestimating “healthy” foods [3], and errors persist even in WFR when ingredients are not fully weighed [4]. Validation studies using doubly-labeled water demonstrate energy intake reporting errors of 20%–50% with substantial interindividual variability [5], highlighting the need for improved methodologies.

Recent artificial intelligence (AI) advances offer promising solutions. Image-assisted methods reduce underreporting by ≤30% when combined with real-time data quality checks [6]. A systematic review of 78 studies revealed convolutional neural networks dominate food recognition systems, used in 76% of studies since 2016 [7]. Early approaches employed classical computer vision with hand-crafted features (Scale-Invariant Feature Transform (SIFT), Speeded-Up Robust Features (SURF), color histograms) combined with traditional classifiers (Support Vector Machines (SVMs), K-Nearest Neighbors (KNN), Artificial Neural Networks (ANNs)), achieving 50%–96% accuracy depending on dataset complexity [7,8]. Deep learning marked a significant leap, with Convolutional Neural Networks (CNNs) learning hierarchical features directly from pixels [7]. Architectures like AlexNet and ResNet achieved ≤90.27% accuracy on Food-101 and 83.15% on UEC-Food 256 datasets (University of Electro-Communications, Japan) [9]. Specialized systems demonstrated varying success: TADA (Technology Assisted Dietary Assessment) achieved 96% classification accuracy on controlled datasets [10], im2calories reported 20% mean absolute errors for calorie estimation on constrained meals [11], and GoCARB showed 12 g mean absolute errors for carbohydrate (CHO) counting [12].

Recent multimodal large language models (LMMs) show promising results. Lo et al. [13] found GPT-4V achieves mean absolute errors comparable with dietitians (46.3 g compared with 48.5 g) even in low-light conditions. Food-specific LLMs like FoodSky leverage contextual information for specialized nutritional guidance and can pass professional culinary examinations [14]. Although commercial applications proliferate (MyFitnessPal, Lose it!, Foodnoms), peer-reviewed evaluations remain limited [7]. Despite advances, challenges persist, including limited dataset diversity, difficulty segmenting mixed dishes, accurate volume estimation from 2D images, and the need for real-world validation [7]. LLM integration presents opportunities and challenges, with concerns about data limitations, potential misinformation, and the need for standardized evaluation methods [15].

### Aim

The aim of this study was to evaluate and compare the performance of 3 leading LLMs (ChatGPT, Claude, and Gemini) in estimating food weight, energy content, and macronutrient content for individual food items and complete meals of different portion sizes, using nutritional database analysis as a reference. Specifically, we aimed to: *1*) assess the accuracy and systematic bias of each model, *2*) compare performance between models for estimating weight (g), energy (kcal), and macronutrients (CHO, protein, and fats); and *3*) evaluate the consistency of estimations across different portion sizes.

## Methods

### Study design

This validation study compared ChatGPT-4o, Claude 3.5 Sonnet, and Gemini 1.5 Pro in estimating nutritional content from standardized food photographs. Using identical prompts, each model analyzed 52 food images (individual components and complete meals in varying portion sizes), with results compared against reference values from direct weighing and nutritional database analysis.

### Food items and reference values

The study included both complete meals (*n* = 12) and individual components (*n* = 16). Complete meals were constructed around 3 starchy bases: rice, pasta, and boiled potatoes. Each base was combined with 1 of 9 protein sources (chicken, scrambled eggs, chicken curry, falafel, lentil curry, minced meat, omelet, patty, or chickpeas) and 1 of 4 vegetables (cabbage, radish, broccoli, or mixed leafy greens including rocket, spinach, and Swiss chard). Additionally, 3 prepackaged convenience meals were included (Pasta Alfredo 400 g, fish and chips 340 g, chicken curry 390 g, Findus Sverige AB). The prepared meals and the starchy components were photographed in 3 portion sizes (small, medium, and large), whereas protein sources and vegetables were photographed in medium portions only. Medium portion sizes were determined based on standard values from The Swedish Food Agency, for example, 115 g boiled pasta or 175 g boiled rice [16]. Small and large portion sizes were defined as 50% and 150% of the medium portion size, respectively. We specifically varied portion sizes of starchy components due to their central role in energy provision and their typically larger contribution to total meal volume. Because starchy foods often represent the most variable component in terms of portion size across different eating contexts and energy needs, the ability of AI models to accurately scale their estimations with increasing portions of these foods is of particular practical importance for dietary assessment applications.

In total, *N* = 52 unique photographs were analyzed. Of these, *n* = 9 depicted meals with only a starchy base, *n* = 9 depicted a protein source, *n* = 4 depicted vegetables, and *n* = 30 showed a complete meal containing starch, a protein source, and vegetables. All meals except the 3 prepackaged items were prepared by the research team specifically for this study. Fresh ingredients were purchased and cooked according to standardized recipes to ensure consistency. Each meal was prepared fresh for photography to maintain visual quality and accurate representation. The 52 photographs represent systematic variations of 12 base dishes, providing sufficient diversity to evaluate model performance across different food types and portion sizes while maintaining experimental control.

All food items were weighed using a calibrated digital scale before photography. For cooked items, weights were recorded after preparation. Energy and macronutrient content were analyzed using the nutrient calculation software package Dietist NET (Kost & Näringsdata), which references the USDA National Nutrient Database (USDA 2022-08-23). For prepackaged meals, manufacturer-provided nutritional information served as reference values. We acknowledge that such information may contain inaccuracies, a limitation inherent to all nutritional research relying on food label data. However, this reflects real-world conditions where AI-based dietary assessment tools would similarly rely on available nutritional databases.

### Photography and scene

**FIGURE 1:**
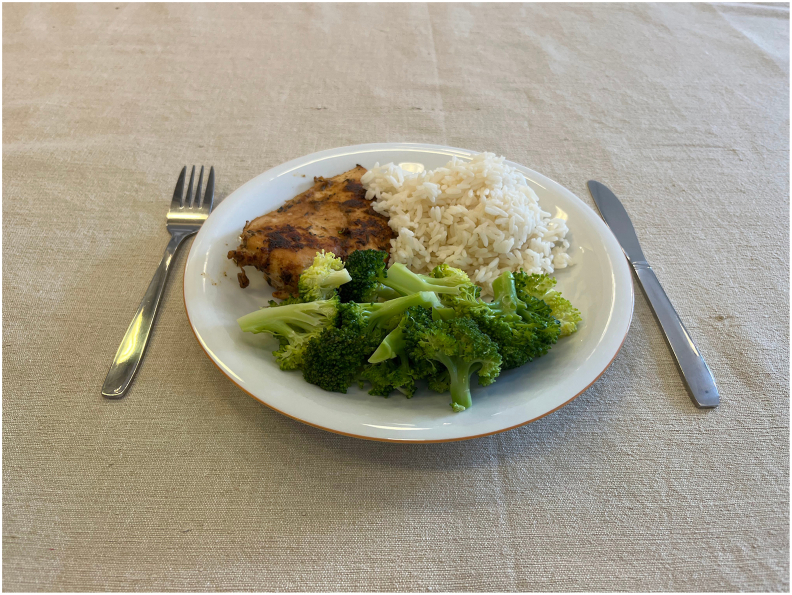
Example of standardized meal photography setup showing a medium-sized portion with rice, grilled chicken breast, and broccoli.

Images were captured using an iPhone 13 dual camera system under standardized conditions. A white porcelain plate (24.3 cm diameter) with beige rim was placed on a beige linen tablecloth (Figure 1). Standard cutlery (19 cm fork, 20.5 cm knife) was positioned 1.5 cm from the plate edge to provide size reference. The photographs were taken from a 42° angle, positioned 20.2 cm above and 20 cm from the plate edge, which is intended to provide the best compromise between displaying both the depth and height of the food [17,18]. For complete meals, vegetables were consistently positioned closest to the camera, with components arranged in distinct sections on the plate. To maintain data quality, fresh portions were prepared for each photograph rather than reusing items.

### AI analysis

Each photograph was independently analyzed by 3 LLMs: ChatGPT-4o (OpenAI, released 2024-05-13), Claude 3.5 Sonnet (Anthropic, released 2024-06-21), and Gemini 1.5 Pro (Google, released 2024-04-09). All models were provided with identical prompts for each image:

“Conduct a nutritional analysis of the different foods in the picture. First, recognize the different components of the dish. Second, estimate the volume of the foods based on their size in relation to other objects in the image. The estimate should not be solely based on typical serving sizes but also consider their size in relation to other objects in the image. Third, determine the nutritional contents of the foods based on typical reference values. Assemble the findings in a table with weight, energy, carbohydrates, fat and protein as columns and the different components of the dish as rows. Finally, summarize the figures as a row at the end.”

The prompt was developed through iterative testing to optimize model performance across different portion sizes. Initial prompts with minimal instructions consistently failed to discriminate between portion sizes, as models defaulted to typical serving assumptions rather than using visual cues. Through systematic testing of various approaches, including detailed volumetric estimation instructions, prompts providing explicit plate dimensions, and prompts instructing models to “act as nutritionists,” we identified key factors affecting performance. Our final prompt was designed to encourage visual assessment over default assumptions, but additional details in the instructions provided no clear benefit. The simpler prompt better reflects real-world usage scenarios where users may not provide extensive contextual information. This standardized approach ensures fair comparison between models.

For each image, the models provided estimates of weight (g), energy content (kcal), and macronutrients (g) for individual components and total meal values. All analyses were conducted independently, with a new chat instance for each image to prevent the models from learning or adapting during the analysis process. All AI analyses were performed during September 2024.

### Statistical analysis

Statistical analyses were performed using R (version 4.4.0). For each model and nutrient (weight, energy, CHO, protein, fat), the following metrics were calculated: mean absolute percentage error (MAPE) with 95% confidence intervals (CIs) using bootstrap resampling (1000 iterations), Pearson correlation coefficients with CIs, and mean differences between AI estimates and reference values. Bland–Altman plots were created to assess agreement between AI estimates and reference values, with data points color-coded by portion size and agreement limits set at ± 1.96 SDs. Systematic bias was evaluated using linear regression of differences against reference values.

Sample size considerations were evaluated post-hoc. With 52 paired observations per model comparison, our design provides 80% power to detect medium effect sizes (*d* ≥ 0.4) at 0.05 significance level. On the basis of the observed variability in energy estimates, this corresponds to detecting systematic differences of ∼60 kcal between models, representing roughly 15% of typical meal energy content.

We chose not to report food identification accuracy as a binary metric due to the complexity of defining “correct” identification in mixed meals. For instance, if a model correctly identified 4 of 5 components but misclassified 1 item, binary classification would mark the entire meal as incorrect, losing nuanced information about partial accuracy. Instead, our focus on continuous nutritional metrics provides more granular insights into model performance.

The manuscript text was edited for clarity and language using the AI-based LLM Claude 3.5 Sonnet (Anthropic). The authors reviewed and verified all AI-assisted edits and take full responsibility for the final content.

## Results

The performance evaluation of ChatGPT, Claude, and Gemini in estimating weight, energy, CHO, protein, and fat content showed varying levels of accuracy across different nutritional components. All specific values and CIs for these analyses are presented in Table 1.

**TABLE 1:** Performance metrics of large language models in dietary assessment.

Nutrient	Model	MAPE (%)	Correlation^1^	Mean bias	Systematic bias
Weight	ChatGPT	36.3 (27.1, 47.3)	0.77 (0.62, 0.86)	7.5 (–22.9, 38.0)	–0.33 (–0.49, –0.17)^1^
Weight	Claude	37.3 (28.4, 46.5)	0.81 (0.69, 0.89)	6.1 (–21.4, 33.7)	–0.30 (–0.44, –0.15)^1^
Weight	Gemini	65.0 (45.1, 88.0)	0.71 (0.54, 0.82)	64.6 (31.5, 97.8)^1^	–0.44 (–0.60, –0.29)^1^
Energy	ChatGPT	35.8 (27.3, 45.1)	0.73 (0.57, 0.84)	–10.0 (–53.0, 33.0)	–0.45 (–0.59, –0.30)^1^
Energy	Claude	35.8 (27.9, 44.7)	0.78 (0.64, 0.87)	–18.9 (–59.0, 21.1)	–0.34 (–0.50, –0.19)^1^
Energy	Gemini	64.2 (45.0, 86.9)	0.63 (0.43, 0.77)	65.0 (12.0, 118.1)^1^	–0.41 (–0.62, –0.20)^1^
CHO	ChatGPT	47.9 (35.9, 61.7)	0.67 (0.49, 0.80)	–1.3 (–7.8, 5.1)	–0.45 (–0.62, –0.28)^1^
CHO	Claude	72.8 (34.9, 141.7)	0.75 (0.60, 0.85)	–0.9 (–6.6, 4.8)	–0.36 (–0.52, –0.20)^1^
CHO	Gemini	66.1 (47.5, 87.3)	0.73 (0.57, 0.83)	9.7 (3.2, 16.1)^1^	–0.23 (–0.44, –0.03)^1^
Protein	ChatGPT	60.7 (42.0, 80.5)	0.73 (0.57, 0.84)	1.8 (–1.3, 5.0)	–0.38 (–0.55, –0.21)^1^
Protein	Claude	61.7 (44.0, 83.1)	0.75 (0.60, 0.85)	1.9 (–1.2, 5.0)	–0.31 (–0.48, –0.14)^1^
Protein	Gemini	109.9 (79.2, 145.6)	0.58 (0.36, 0.73)	7.3 (3.4, 11.3)^1^	–0.50 (–0.70, –0.30)^1^
Fat	ChatGPT	51.8 (38.1, 70.7)	0.65 (0.45, 0.78)	–1.4 (–4.0, 1.2)	–0.28 (–0.52, –0.04)^1^
Fat	Claude	41.7 (32.4, 53.1)	0.72 (0.55, 0.83)	–2.1 (–4.4, 0.1)	–0.25 (–0.46, –0.04)^1^
Fat	Gemini	89.6 (46.9, 152.1)	0.64 (0.45, 0.78)	1.2 (–1.6, 4.0)	–0.20 (–0.47, 0.07)

Values are presented as an estimate (95% confidence interval).

Abbreviations: AI, artificial intelligence; CHO, carbohydrates; MAPE, mean absolute percentage error.

1 Significantly different from null. All correlations were statistically significant (*P* < 0.05). Mean bias values represent AI estimate minus reference value. Systematic bias represents the slope of the regression line of differences against reference values.

**FIGURE 2:**
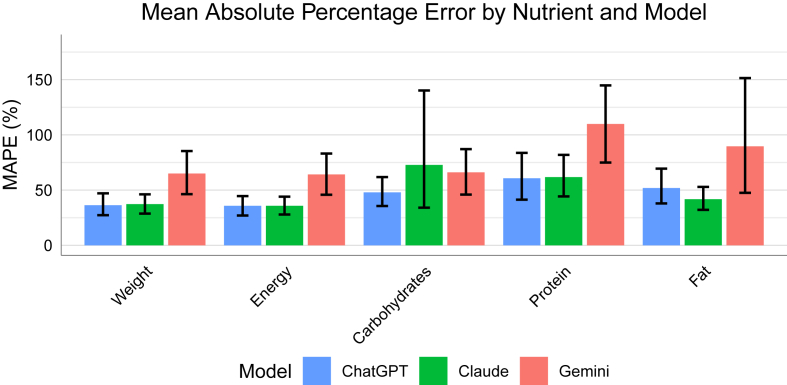
Mean absolute percentage error (MAPE) by nutrient and model. Error bars represent 95% confidence intervals calculated using bootstrap resampling with 1000 iterations.

MAPE, as shown in Figure 2, demonstrated that ChatGPT and Claude achieved similar accuracy levels for weight (36.3% and 37.3%, respectively) and energy (both 35.8%). However, for CHO, Claude showed notably higher error (72.8%) compared with ChatGPT (47.9%). Both models performed similarly for protein estimation (60.7% compared with 61.7%), whereas Claude showed better accuracy for fat (41.7% compared with 51.8%). Gemini consistently demonstrated higher error rates across all nutrients, with MAPE values ranging from 64.2% for energy to 109.9% for protein.

Correlation analysis between model estimates and reference values demonstrated moderate to strong positive relationships across all nutrients. Claude achieved the highest overall correlations (*r* = 0.72–0.81), followed closely by ChatGPT (*r* = 0.65–0.77). For weight and energy content, both models showed strong correlations (*r* > 0.73), whereas correlations for macronutrients were somewhat lower. Gemini showed the weakest correlations across all measures, particularly for protein (*r* = 0.58).

Direct comparison between models revealed no statistically significant differences between ChatGPT and Claude in MAPE values for most nutrients (*P* > 0.15), except for fat estimation, where Claude performed significantly better (*P* = 0.04). Both models significantly outperformed Gemini for weight, energy, and protein estimation (all *P* < 0.01). The correlation coefficient differences between models were generally not significant, with exceptions only for select comparisons between Gemini and the other models.

**FIGURE 3:**
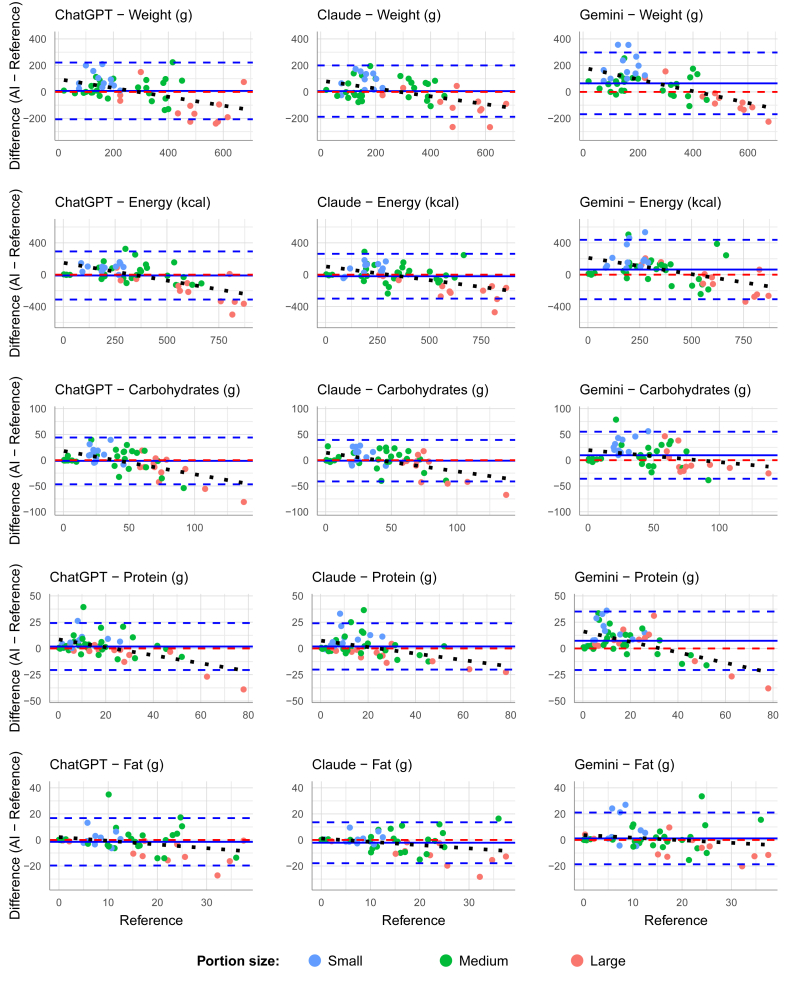
Bland–Altman plots comparing AI model estimates to reference values for weight, energy, and macronutrients. Plots show the difference between AI estimates and reference values (*y*-axis) against reference values (*x*-axis). Solid blue line represents mean bias, dashed red line indicates zero difference, dashed black line shows systematic bias trend, and dashed blue lines indicate ± 1.96 SD limits of agreement. AI, artificial intelligence.

Bland–Altman analysis, illustrated in Figure 3, revealed varying patterns of bias. Gemini showed significant positive mean biases for weight (+64.6 g), energy (+65.0 kcal), and other nutrients, whereas ChatGPT and Claude demonstrated smaller, nonsignificant biases (weight: +7.5 g and +6.1 g, respectively). Systematic bias analysis showed significant negative slopes for most model-nutrient combinations, indicating a portion size-dependent bias.

Further analysis of portion size effects revealed that all models demonstrated decreasing accuracy as portion size increased. This trend was most evident in the Bland–Altman plots, where small portions (shown in blue) typically clustered near the zero-difference line, whereas large portions (shown in green) exhibited greater variability and more extreme underestimation. The systematic bias slopes ranged from –0.23 to –0.50 across nutrients and models, with ChatGPT showing the strongest portion size dependency for CHO (–0.45) and Gemini for protein (–0.50). Relative accuracy was consistently better for small portions, where MAPE values were ∼20%–30% lower than for large portions across all models.

A couple of illustrative examples are worth mentioning. Gemini misidentified falafel as meatballs in 1 image, resulting in a 360% overestimate of protein due to the vastly different protein densities of these foods. Similarly, Claude misidentified scrambled eggs as a pasta dish in 1 image, resulting in a 1788% overestimate of CHO and highly influencing the MAPE for Claudes estimates of CHO, as seen in Figure 2. Another example is a large portion of a lentil curry dish, where ChatGPT estimated a weight of 255 g compared with the actual weight of 480 g, which influenced the estimate of all nutrients.

## Discussion

This study provides the first systematic evaluation of multiple LMMs for nutritional content estimation from food images of different portion sizes. ChatGPT and Claude achieved MAPE values of ∼36% for weight and energy estimation, whereas Gemini showed substantially higher errors across all nutrients.

ChatGPT and Claude's comparable performance suggests practical utility thresholds. Their accuracy aligns with traditional self-reported methods, where doubly-labeled water validation shows energy intake reporting errors of 20%–50% [5]. Unlike traditional methods subject to recall bias, social desirability effects, and participant burden, AI-based estimations operate independently of these human factors, offering advantages for continuous passive monitoring.

All models exhibited consistent underestimation as portion size increased, particularly relevant for athletic populations with high energy demands [19]. Comparing with 4 studies validating estimated diet records against doubly-labeled water in weight-stable athletes (Supplemental Table 1), MAPE was 26.5% ± 16.8% (95% CI: 20.3%, 32.8%). Our evaluation showed MAPE of 35.8% for ChatGPT and Claude, and 64.2% for Gemini—suggesting the former 2 perform comparably with traditional methods whereas Gemini produces markedly larger errors. Importantly, AI-based estimations operate without user burden or reporting fatigue, offering promise for scalable monitoring.

AI models demonstrate inherent output variability, identical prompts won't necessarily return identical responses due to the stochastic nature. Although introducing uncertainty, this enables multiple analyses to generate CIs. Future applications might perform multiple inferences reporting ranges rather than point estimates. Clinical applications could establish CIs for reliability assessment; research applications might average inferences while providing uncertainty measures. This offers opportunities for nuanced assessment, acknowledging inherent uncertainty.

Recent research confirms AI potential for dietary assessment, though challenges remain in obtaining in-depth information from 2D images [20]. Negative systematic bias slopes (–0.23 to –0.50) indicate models struggle scaling estimations with increasing portions. This limitation was pronounced in our design, where consistent vegetable placement in front, while standardizing photography, created systematic bias, worsening with portion size. As starchy bases and protein sources positioned behind vegetables became increasingly obscured with larger portions, models had progressively less visual information about primary calorie-contributing components.

Macronutrient estimation proved challenging. Protein estimation exceeded 60% MAPE, possibly due to difficulty distinguishing protein-rich components or estimating density visually. High variability across protein sources compounds this; correctly identifying animal compared with plant protein causes several-fold differences, exemplified when Gemini misidentified falafel as meatballs (360% protein overestimate). Claude's high CHO MAPE (72.8%) resulted from misidentifying scrambled eggs as pasta (1788% overestimation), highlighting how misidentifications disproportionately affect macronutrient estimates.

Performance differences likely stem from variations in training data, architecture, and optimization strategies. Gemini's consistently poorer performance might reflect less extensive food-related training or different multimodal integration. ChatGPT and Claude's similar performance suggests convergent capabilities among leading LLMs.

Our design could be enhanced through elaborate prompting. Instructing models to estimate dimensions before calculating volume might improve accuracy. We deliberately excluded plate/cutlery dimensions to simulate real-world scenarios, potentially limiting accuracy. Recent research demonstrates sophisticated prompting techniques enhance LLM nutrition accuracy, including chain-of-thought for volumetric estimation or retrieval augmented prompting [21]. Providing contextual information could help make accurate assumptions about servings and preparation. Research shows LLMs with crafted instructions and appropriate context produce dietitian-standard explanations [13,22]. However, our approach ensures methodological rigor in model comparison and establishes baseline capabilities under standardized conditions.

Compared with narrow AI models trained for food recognition using CNNs, LLMs offer unique advantages utilizing environmental cues and reference objects. Their broad training enables understanding contextual relationships between cutlery, plates, and food, potentially providing more robust size estimation [13]. However, LMMs require more computational resources, preventing local operation and highlighting integrity problems because photographs must be uploaded to data centers.

Moderate to strong correlations (*r* = 0.58–0.81) indicate reasonable relative accuracy despite limited absolute accuracy, suggesting utility for tracking dietary patterns even if absolute values require calibration. Error consistency implies systematic correction factors could improve accuracy, though these would need portion size dependence.

All evaluated models represent mid-2024 state-of-the-art. The AI landscape is rapidly evolving—OpenAI introduced Large Reasoning Models with o1, using reinforcement learning for complex reasoning through chain-of-thought processes [23]. These models, released late 2024/early 2025, could address systematic errors through step-by-step visual analysis, decomposing complex tasks: identifying foods, estimating dimensions, calculating volumes, and applying nutritional databases systematically.

Several limitations warrant consideration: static images from fixed angles compared with multiple angles or video; relatively simple presentations compared with real-world complex mixtures; standard configurations without food recognition fine-tuning. Domain-specific knowledge and specialized training significantly improve LLM food science performance [15]. Future research should explore depth-sensing technologies, food-specific LLMs, or fine-tuning on comprehensive datasets. Food-oriented LLMs demonstrate better domain-specific understanding [14]. Combining AI with minimal user input could substantially improve accuracy while maintaining feasibility [13].

The evolving landscape raises standardization considerations. As technologies mature, establishing benchmarks becomes crucial for comparing systems. Our study provides a controlled evaluation framework for future refinement.

In conclusion, current general-purpose LLMs show promise for dietary assessment. ChatGPT and Claude demonstrate performance approaching traditional methods without user burden, suggesting utility as screening tools. However, systematic underestimation of large portions and high macronutrient variability indicate unsuitability for precise assessment in athletic populations requiring accurate quantification. Continued advancement, particularly addressing portion size challenges and potentially through reasoning models, remains necessary before AI-based assessment revolutionizes nutritional monitoring in sports science.

## Author contributions

The authors’ responsibilities are as follows – JF, SP: designed research and wrote article; ES, MT: conducted research; JF: analyzed data and had primary responsibility for final content; and all authors: read and approved the final manuscript.

## Data availability

Data described in the manuscript, code book, and analytic code will be made available on request pending approval by the corresponding author.

## Declaration of generative AI and AI-assisted technologies in the writing process

During the preparation of this work, the author(s) used Claude 3.5 Sonnet (Anthropic) to edit text for clarity and language and to draft the abstract. After using this tool/service, the author(s) reviewed and edited the content as needed and take(s) full responsibility for the content of the publication.

## Funding

The authors reported no funding received for this study.

## Conflict of interest

The authors declare that they have no known competing financial interests or personal relationships that could have appeared to influence the work reported in this article.
