# Supplementary material for: Performance Evaluation of 3 Large Language Models for Nutritional Content Estimation from Food Images
Source: Curr Dev Nutr. 2025 Sep 9;9(10):107556. doi: 10.1016/j.cdnut.2025.107556 (PMC12513282; doi:10.1016/j.cdnut.2025.107556)
Supplement: multimedia component 1 [file mmc1.pdf]

Supplementary Materials

Summary of selected studies (N=4) reporting mean absolute percentage error (MAPE) in energy intake compared to total energy expenditure (TEE) measured by the doubly labeled water method. Energy intake (EI) was assessed using estimated (est.) diet records. Studies were included based on the criterion that participants maintained stable body weight and/or body composition during the monitoring period, indicating a state of energy balance. Thus, discrepancies between EI and TEE were interpreted as under-recording rather than under-eating. MAPE was calculated from individual-level differences between reported EI and measured EE, expressed as the MAPE for each participant. The table presents respective study's dietary assessment method, athlete population, sample size, and respective study's MAPE with standard deviation and 95% confidence intervals (CI).

Supplementary Table 1.

Study	Dietary assessment method	Athletes	MAPE ± SD % (95% upper – lower CI)
Edwards JE, Lindeman AK, Mikesky AE, Stager JM. Energy balance in highly trained female endurance runners. Med Sci Sports Exerc 1993; 25:1398–1404.	7-d est.	Distance runners (9 females)	29.8 ± 17.9 (16.1 – 43.5)
Trappe TA, Gastaldelli A, Jozsi AC, et al. Energy expenditure of swimmers during high volume training. Med Sci Sports Exerc 1997; 29:950–954.	2-d est.	Swimmers (5 females)	44.8 ± 4.9 (38.6 – 50.9)
Ebine N, Feng JY, Homma M, et al. Total energy expenditure of elite synchronized swimmers measured by the doubly labeled water method. Eur J Appl Physiol 2000; 83:1–6.	2-d est.	Synchronized swimmers (9 females)	23.6 ± 15.7 (11.5 – 35.7)
Ebine N, Rafamantanantsoa HH, Nayuki Y, et al. Measurement of total energy expenditure by the doubly labelled water method in professional soccer players. J Sports Sci 2002; 20:391–397.	7-d est.	Professional soccer players (7 males)	13.2 ± 8.8 (5.1 – 21.4)
Weighted mean, all studies (N=4)		N=30 participants	26.6 ± 16.8 (20.2 – 32.8)
